# Association between Sleep Duration and Subclinical Thyroid Dysfunction Based on Nationally Representative Data

**DOI:** 10.3390/jcm8112010

**Published:** 2019-11-18

**Authors:** Woojun Kim, Jeongmin Lee, Jeonghoon Ha, Kwanhoon Jo, Dong-Jun Lim, Jung-Min Lee, Sang-Ah Chang, Moo-Il Kang, Min-Hee Kim

**Affiliations:** 1 Department of Neurology, Seoul St. Mary’s Hospital, College of Medicine, The Catholic University of Korea, Seoul 06591, Korea; wjkim@catholic.ac.kr; 2 Division of Endocrinology and Metabolism, Department of Internal Medicine, Eunpyeong St. Mary’s Hospital, College of Medicine, The Catholic University of Korea, Seoul 03312, Korea; 082mdk45@gmail.com (J.L.); leejm68@catholic.ac.kr (J.-M.L.); sangah@catholic.ac.kr (S.-A.C.); 3 Division of Endocrinology and Metabolism, Department of Internal Medicine, Seoul St. Mary’s Hospital, College of Medicine, The Catholic University of Korea, Seoul 06591, Korea; hajhoon@catholic.ac.kr (J.H.); ldj6026@catholic.ac.kr (D.-J.L.); mikang@catholic.ac.kr (M.-I.K.); 4 Division of Endocrinology and Metabolism, Department of Internal Medicine, Incheon St. Mary’s Hospital, College of Medicine, The Catholic University of Korea, Seoul 21431, Korea; lovi@naver.com

**Keywords:** Korea National Health and Nutrition Examination Survey (KNHANES), sleep, thyroid function test, thyroid disease, subclinical hyperthyroidism, subclinical hypothyroidism

## Abstract

Background: Sleep duration is an identified risk factor for adverse health outcomes. As the endocrine system is closely intertwined with sleep duration and quality, the association between endocrine dysfunction and sleep has been evaluated. Thyroid function, particularly that related to thyrotropin (TSH), is also known to be influenced by the sleep/awake status and circadian rhythm. Additionally, a link between sleep duration and autoimmunity, which is a common cause of thyroid dysfunction, has been suggested; however, depending on the sleep deprivation method used in studies, the effects of sleep on thyroid function vary. The relationship between subclinical thyroid dysfunction and sleep duration is poorly documented. Thus, to elucidate the impact of sleep on thyroid function, we investigated the association of subclinical thyroid dysfunction with sleep duration using representative data from the sixth Korea National Health and Nutrition Examination Survey, conducted from 2013 to 2015. Methods: In all, 4945 participants (2543 male and 2402 female) were included after excluding subjects using the following criteria: <19 years of age, free T4 level outside the normal range, history of thyroid disease, or incomplete data. The population was classified into three groups: short sleeper (<7 h/day), normal sleeper (7–8 h/day), and long sleeper (>8 h/day). The odds ratio (OR) for subclinical hypothyroidism or hyperthyroidism according to sleep duration was evaluated. Results: The short, normal, and long sleeper groups consisted of 2097, 2514, and 334 subjects, respectively. On multiple logistic regression analysis, compared to normal sleepers, short sleepers showed a significantly increased risk of subclinical hyperthyroidism (OR 1.37, 95% confidential interval (CI) 1.02–1.84, *p* = 0.036), while the risk of subclinical hypothyroidism in short sleepers was not elevated. Comparing long sleepers to normal sleepers, the OR for subclinical hyperthyroidism and hypothyroidism was 1.79 (95% CI 1.12–2.86, *p* = 0.015) and 1.91 (95% CI 1.03–3.53, *p* = 0.039), respectively. Conclusions: Both shorter and longer sleep durations were associated with an increase in the risk of subclinical thyroid dysfunction compared to the optimal sleep duration. This analysis of representative population data shows that sleep duration could intertwine with thyroid function resulting in increased risk of subclinical thyroid dysfunction.

## 1. Introduction

There are concerns and debates regarding adverse health outcomes caused by disrupted sleep and decreased sleep duration [1], both of which are prevalent in modern society. In this context, the association of endocrine dysfunction with sleep is intriguing because the endocrine system is closely intertwined with sleep duration and quality. Sleep could influence hormone secretion, and endocrine dysfunction can also affect sleep. There are two processes that link sleep and endocrine function: the circadian rhythm and the sleep/wake state, both of which affect the secretion of hormones. Adrenocorticotropic hormone and cortisol are well-known hormones mainly regulated by the circadian rhythm [2], and growth hormone secretion is mainly regulated by the sleep/wake state [3].

Thyroid function is mainly controlled by the hypothalamic–pituitary–thyroid axis. Thyrotropin (TSH), tetraiodothyronine (T4), and triiodothyronine (T3) comprise the feedback loop for maintaining the homeostasis of thyroid function. Among these hormones, TSH is best known to be primarily influenced by the circadian rhythm [4,5,6,7]. There is a steep increase in TSH in the early evening, reaching a plateau at approximately 2–3 a.m. Thereafter, the serum TSH level decreases until the early evening. Free T3 also has a circadian cycle that parallels that of TSH with a delayed pattern [5]. In terms of how sleep influences thyroid function, several studies have shown the effects of sleep deprivation on TSH secretion [4,8,9,10,11,12]. Sleep deprivation has shown inconsistent effects on TSH secretion depending on the pattern of sleep deprivation (short-term or long-term sleep deprivation, duration of sleep deprivation). Although acute, extreme deprivation has been shown to increase the secretion of TSH and augment the surge of TSH release, long-term, modest sleep deprivation suppresses the secretion of TSH [11,12]. However, most of the aforementioned findings are from studies of only dozens of subjects.

In addition to the influence of the circadian rhythm and sleep–wake status on thyroid dysfunction, an association of sleep duration with thyroid function could be postulated according to possible crosstalk between sleep duration and immunity [13,14]. In fact, an increase in the risk [15,16,17] and activity [18,19,20] of several autoimmune diseases has been shown to be related to sleep duration. Considering that altered autoimmunity is a common cause of thyroid dysfunction, there is the possibility of a relationship between thyroid dysfunction and sleep duration.

No studies have demonstrated a relationship between sleep duration and thyroid function in a large population. In addition, as subtle changes in the TSH concentration do not necessarily correlate with clinical implications, it would be clinically significant to investigate the relationship between sleep duration and subclinical thyroid dysfunction, which is biochemically determined and supposed to be managed in clinical practice regardless of treatment initiation. Thus, the aim of this study was to identify the association of subclinical thyroid dysfunction with sleep duration using representative data from the Korean population.

## 2. Materials and Methods

### 2.1. Study Population and Data Collection

The study data were extracted from the sixth Korea National Health and Nutrition Examination Survey (KNHANES VI, 2013–2015). The KNHANES has been conducted by the Ministry of Health and Welfare of the Korean Government since 1998 to assess the general health and nutritional status of Koreans. Initially, 22,948 adults were first identified from KNHANES VI. From this group, individuals were excluded based on the following criteria: no test for free T4 and TSH (*n* = 15,887), age less than 19 years (*n* = 1021), reported history of thyroid disease (*n* = 179), no data on urine iodine levels (*n* = 319), no data on sleep duration (*n* = 319), incomplete data (*n* = 215), or free T4 level outside the normal range (0.89–1.76 ng/dL) (*n* = 119). After the exclusion process, 4945 participants remained eligible for the final analysis. Blood and urine samples were drawn in the morning after more than 8 h of fasting. All samples were acquired before 12 pm. Laboratory tests for serum free T4, TSH, and urine iodine were performed in stratified subsampling according to sex and age in KNHANES VI.

Data relating to a prior history of thyroid disease, use of medications that could affect thyroid function, and family history of thyroid disease were collected by interview. Laboratory measurements of the serum TSH, free T4, anti-thyroid peroxidase (TPO) antibody, and urine iodine levels were obtained using subsampling stratified by sex and age, as previously described [21]. Briefly, TSH, free T4, and anti-TPO antibody levels were measured using an E-TSH kit (Roche Diagnostics, Basel, Switzerland), an E-Free T4 kit (Roche Diagnostics, Basel, Switzerland), and an E-Anti-TPO kit (Roche Diagnostics, Basel, Switzerland), respectively. The urine iodine level was measured by inductively coupled plasma mass spectrometry (PerkinElmer; Waltham MA, USA). To estimate the urine iodine status accurately, the urine iodine creatinine ratio (µg (iodine)/g (creatinine)) was calculated.

In the KNHANES, written informed consent was obtained from every participant prior to completion of the survey, and secondary anonymized data were used for analysis, as previously described. The study protocol was approved by the Institutional Review Board (IRB approval no. KC17ZESI0571) of the Catholic University of Korea, Seoul, Korea.

### 2.2. Sleep Duration and Thyroid Function

Sleep duration was obtained from the self-reported questionnaire using the question ‘How many hours of sleep do you usually get in a day on average?’ We classified subjects into three groups by sleep duration according to previous studies [22,23,24]: short sleeper (<7 h/day), normal sleeper (7–8 h/day), and long sleeper (>8 h/day). For detailed subgroup analysis, subjects were also divided into five groups: extreme short (<5 h/day), mild short (5–6 h/day), normal (7–8 h/day), mild long (9–10 h/day), and extreme long (≥10 h/day). All blood samples were collected in the morning before 12 pm (when serum TSH levels are relatively stable and free from surge) after 8 h of fasting. According to recent reference TSH ranges based on Korean population data [25], subjects were categorized into the following groups by TSH level: euthyroidism (0.62–6.68 mIU/L), subclinical hyperthyroidism (<0.62 mIU/L), and subclinical hypothyroidism (>6.68 mIU/L). The subjects were also grouped by anti-TPO antibody level, as follows: negative (<34 IU/mL) and positive (≥34 IU/mL).

### 2.3. Other Variables

The following covariates were obtained using a standardized questionnaire in the KNHANES: age, sex, body mass index (BMI, weight in kilograms divided by the square of height in meters), smoking status, alcohol intake, household income, educational level, and regular exercise. We divided the subjects according to BMI into four groups based on Asian criteria for obesity: <18.5, 18.5–23, 23–25, and ≥25 [26]. The subjects were stratified by educational level into four groups: ≤6 y, 7–9 y, 10–12 y, and >12 y of education. The subjects were stratified by the average monthly household income into four groups: ≤1000, 1001–2000, 2001–3000, and ≥3001 (thousand KRW). Smoking status was determined by the presence of current smoking. Alcohol consumption was categorized as no consumption, <4 times per month, and ≥4 times per month. Regular exercise was defined as strenuous physical activity for more than 20 min per day at least three days per week, moderate physical activity for more than 30 min at least five days per week, or walking for more than 30 min at least five days per week. The working pattern was categorized as: daytime, evening, nighttime, or irregular working.

### 2.4. Statistical Analysis

For comparison of the baseline characteristics, the Rao–Scott chi-squared test, ANOVA or *t*-test was used, as appropriate. Student’s *t*-test and ANOVA were used to compare the mean TSH level among the groups, as appropriate. The Rao–Scott chi-squared test and *t*-test were performed to evaluate the thyroid function status and the mean TSH value according to sleep duration. Odds ratios (ORs) for subclinical hypothyroidism or hyperthyroidism according to sleep duration were evaluated by logistic regression analysis. In addition to demographic variables, such as age and sex, variables that showed significant differences between groups were considered confounding factors. The regression coefficient (B) and 95% confidence interval (CI) were also calculated; *p* < 0.05 (two-tailed) was considered significant. SPSS 17.0 for Windows (SPSS, Inc., Chicago, IL, USA) was used to perform all statistical analyses.

## 3. Results

### 3.1. Baseline Characteristics

The demographic characteristics of the study population are shown in Table 1. The study cohort consisted of 2543 males and 2402 females. There were 2097 short sleepers, 2514 normal sleepers, and 334 long sleepers. While long sleepers were younger (41.17 ± 1.09 years) than normal sleepers (43.46 ± 0.32 years), short sleepers were older (46.71 ± 0.35 years). The short sleepers group showed the highest mean BMI (24.22 ± 0.09). Significant differences in the income/educational levels, prevalence of diabetes, and urine iodine level were observed among the three groups (Table 1). Upon comparison of short or long sleepers with normal sleepers, short sleepers showed a higher BMI, more frequent alcohol consumption, and lower income/educational levels than did normal sleepers. Long sleepers presented a lower BMI, lower income/educational levels, lower urine iodine levels, and a higher frequency of diabetes than did normal sleepers. In long sleepers, the prevalence of daytime working (78.98%) was the lowest (85.88% in short sleepers and 85.56% in normal sleepers).

### 3.2. Mean TSH and Prevalence of Subclinical Thyroid Dysfunction According to Sleep Duration

Subclinical thyroid diseases were more prevalent in short sleepers (6.90%) and long sleepers (8.51%) than in normal sleepers (4.94%) (*p* = 0.0399) (Table 2). However, although long sleepers showed the highest mean TSH value (2.85 ± 0.14), only borderline statistical significance was observed in multiple comparisons (*p* = 0.0688 for long vs. normal sleepers, *p* = 0.0607 for long vs. short sleepers).

### 3.3. Risk of Subclinical Thyroid Dysfunction According to Sleep Duration

In the multiple logistic regression analysis, short sleepers showed a significantly increased risk of subclinical hyperthyroidism compared to normal sleepers (Table 3). Included confounding factors were age, sex, BMI, diabetes, alcohol consumption, household income level, education, urine iodine creatinine ratio, and working pattern. Statistical significance was evident after adjusting for confounding factors (OR 1.38 (1.02–1.85), *p* = 0.034 in model 1; OR 1.37 (1.02–1.84), *p* = 0.036 in model 2; and OR 1.35 (1.00–1.82), *p* = 0.048 in model 3, respectively). In contrast, the risk of subclinical hypothyroidism in short sleepers was not elevated compared to that in normal sleepers. In long sleepers, the risk of subclinical hyperthyroidism was significantly elevated. After adjusting for confounding factors, the OR for subclinical hyperthyroidism was 1.77 (1.11–2.84) in model 1 (*p* = 0.0175), 1.79 (1.12–2.86) in model 2 (*p* = 0.0154) and 1.77 (1.11–2.84) in model 3 (*p* = 0.0169). Unlike in short sleepers, after adjusting for all possible confounding factors, long sleepers exhibited a significantly increased risk of subclinical hypothyroidism, with an OR of 1.91 (1.03–3.53) (*p* = 0.0399) in model 2 and an OR of 1.89 (1.02–3.49) (*p* = 0.0441) in model 3. In the subgroup analysis of subjects who worked evenings or nights, no increased risk of subclinical thyroid dysfunction was found in short or long sleepers compared to normal sleepers (Supplementary Table S1). When the sleep duration was subdivided into extreme short (<5 h) (*n* = 180), mild short (5–6 h) (*n* = 1917), normal (7–8 h) (*n* = 2514), mild long (9–10 h) (*n* = 316), and extreme long sleepers (>10 h) (*n* = 18), an increased risk of subclinical hyperthyroidism was observed in extreme short and mild long sleepers (Supplementary Table S2). Subclinical hypothyroidism was significantly increased only in mild long sleepers (model 2).

Additionally, because anti-TPO antibody positivity has a substantial influence on thyroid function, subgroup analysis was performed in subjects who were negative for anti-TPO antibody (Table 4). In terms of subclinical hyperthyroidism, the risk was elevated in both short and long sleepers after analysis of the total population. Interestingly, the statistical significance of the increased risk of subclinical hypothyroidism in long sleepers was more prominent (Table 4) compared to that in the analysis of the total population.

## 4. Discussion

When stratified by sleep duration, the risk of mild thyroid dysfunction was estimated differently. The risk of subclinical hyperthyroidism was increased in both short sleepers and long sleepers compared to that in normal sleepers. These findings were maintained after adjusting for possible confounding factors. However, the risk of subclinical hypothyroidism in short sleepers was similar to that in normal sleepers, while long sleepers showed a higher incidence of subclinical hypothyroidism than normal sleepers after adjusting for possible confounding factors. These findings became prominent when an analysis was performed only in subjects with anti-TPO antibody negativity.

Previously, several studies have evaluated the effect of sleep on thyroid function. Circadian changes in TSH secretion [4,5,6,7,27] and T3 secretion parallel to the TSH circadian rhythm [5,28] have been observed. In contrast, free T4 does not show a definitive circadian rhythm [5], which is correlated with TSH rhythmicity; this is possibly due to the long half-life of free T4 (approximately 6.7 days). The influence of sleep has also been studied in many ways for several decades. A summary of studies from 1970 to 1990 showed that sleep deprivation was associated with further TSH secretion [29]. Most older studies in the literature implemented interventions with extreme, short-term sleep deprivation to evaluate the effect of sleep on the TSH circadian rhythm. In these sleep deprivation studies, increased TSH secretion and augmentation of the TSH surge were observed. In contrast, more recent studies [11,12] have evaluated the effects of sleep on TSH secretion by relatively long-term, modest sleep deprivation. In these studies, TSH suppression in response to sleep deprivation was observed repeatedly. However, in these previous studies, regardless of the mode of sleep deprivation, only a small number of subjects were recruited [4,8,9,10,11,12]. Therefore, it would be intriguing to elucidate the effect of sleep on thyroid function in a large population, especially using nationally representative data.

In addition to the effects of the circadian rhythm and sleep/wake status on thyroid function, crosstalk between autoimmunity and sleep duration [13,14] could raise the possibility of the sleep duration affecting thyroid function. Although not well studied in thyroid autoimmune disease, an increased risk of several autoimmune diseases, such as systemic lupus erythematosus (SLE), rheumatic arthritis, and ankylosing spondylitis, has been reported in certain populations [15,17,30]. Some clinical studies have also observed an association of autoimmune disease activity with sleep [18,20]. Additionally, studies of animal models of SLE have demonstrated a certain role of sleep deprivation in the development of the disease [16] and an association of sleep with increased disease activity [19]. Therefore, alterations in thyroid function caused by primary autoimmune thyroid diseases might be related to sleep duration.

Based on our results, both shortened and prolonged sleep durations affect the risk of subclinical thyroid dysfunction, which is an alteration in the serum TSH level beyond the normal range. Although the underlying mechanism for the association between sleep duration and subclinical thyroid dysfunction could not be easily deduced or confirmed based on our findings, several explanations for the phenomenon can be suggested.

First, as shown in previous studies evaluating the effect of sleep deprivation, sleep duration could affect TSH secretion, resulting in TSH suppression (subclinical hyperthyroidism) or stimulation (subclinical hypothyroidism). In this context, the increased risk of subclinical hyperthyroidism in short sleepers found by this study is supported by the findings of studies evaluating the effects of modest but relatively long-term sleep deprivation on the TSH circadian rhythm, resulting in the suppression of TSH secretion [11,12]. Considering that sleep duration in our study was reported as an average time, we could assume that our results would be comparable to the results of studies on modest and long-term sleep deprivation rather than the results of those on extreme and short-term deprivation. A high probability of TSH suppression in short sleepers would lead to an elevated risk of subclinical hyperthyroidism. Meanwhile, the increased risk of subclinical hyperthyroidism in long sleepers is not fully explained in this way. Furthermore, there was a significant association of subclinical hypothyroidism (increased TSH secretion) with a long sleep duration. One possible rationalization for the various effects of sleep duration on the existence of subclinical thyroid dysfunction stems from differences in sleep initiation and termination times. Regardless of the effect of sleep, TSH is regulated by the circadian rhythm; the concentration sharply increases starting in the early evening, peaks at 2–3 a.m., and decreases until the afternoon. Thus, despite individuals reporting the same sleep duration, the effects of sleep (especially prolonged sleep) on the magnitude of TSH secretion might be different according to an individual’s sleep initiation and termination times. For example, the effect of sleep on TSH secretion would be different between individuals who go to sleep between 8 p.m. and 3 a.m., when TSH secretion is the highest during the 24 h period, and individuals who go to sleep between 2 and 8 a.m., when TSH secretion begins to decrease. In fact, in a subgroup analysis of evening and nighttime workers who could not sleep during the night, when TSH surge occurs, sleep duration has minimal effects on the risk of subclinical thyroid dysfunction (Supplementary Table S1). However, this cannot fully explain the phenomenon because statistical significance was maintained after adjusting for working pattern, which might be a main determinant of sleep initiation and termination. As the effect of prolonged sleep in the long term has rarely been evaluated previously, the possibility that a long sleep duration plays a role in both the suppression and stimulation of TSH secretion cannot be excluded.

Second, beyond the effects of the circadian rhythm and sleep–wake status on thyroid function, the interaction between sleep and the immune system could provide a plausible hypothesis for the increased risk of subclinical thyroid dysfunction in subjects with an inadequate sleep duration. An association of sleep disorder with the risk of autoimmune diseases, including rheumatoid arthritis, ankylosing spondylitis, and SLE, has also been reported [15]. Thus, a relationship between sleep disturbance and thyroid dysfunction, which is caused mainly by autoimmune processes, could be suspected. However, as our results became robust after the exclusion of subjects positive for anti-TPO antibody, a major thyroid autoantibody, an explanation based on crosstalk between sleep and autoimmune thyroid disease would be limited.

Third, individual susceptibilities to the impact of sleep on TSH secretion or the development of subclinical thyroid disease should be considered because we could not find changes in the mean TSH. Hypothetically, there might be interindividual variation in the TSH change in response to sleep duration. However, there are no studies to support this hypothesis. Further studies would be required from the perspective of interindividual variability in thyroid function changes in response to sleep duration.

Regarding a normal sleep duration, there have been numerous studies on sleep duration and various diseases and mortality: A short sleep duration is associated with hypertension [31]; short and long sleep durations are associated with an increased risk of type 2 diabetes [22]; short and long sleep durations are associated with cardiovascular disease-related mortality [23]; a short sleep duration is associated with elevated metabolic syndrome among men, while a long sleep duration is associated with metabolic syndrome in both men and women [24]; and a long sleep duration is associated with increased mortality [32]. Referring to those studies, we defined 7–8 h as a normal sleep duration, which was found to be optimal for reducing the risk of subclinical thyroid disease.

Several limitations could be noted in the current study. First, thyroid function was measured only once. Second, because the sampling time could affect the serum TSH level [33], the lack of a specific time for each sampling could hinder the interpretation of the results. However, as all blood samples were drawn in the morning before 12 pm when TSH levels are relatively stable, we could assume that there was minimized effect of blood sampling time on our results. Third, the sleep duration was based on the subject’s recall and may not reflect the exact sleep duration. The questionnaire for evaluating sleep duration in the KNHANES was not developed for this specific study. However, we could assume that the average sleep duration was provided based on individual recall. Fourth, sleep quality was not included in the analysis. Fifth, as results of the study were based on cross-sectional design, causal relationship would not be clear. Thus, though we suggested several mechanisms for the phenomenon, the possibility of thyroid function causing the sleep pattern changes should be also considered. Finally, it could not be evaluated whether the observed phenomenon, i.e., alterations in thyroid function related to sleep duration, is associated with adverse long-term health consequences.

In conclusion, subclinical thyroid dysfunction, including subclinical hyperthyroidism and hypothyroidism, is related to sleep duration. Both shorter and longer than optimal sleep durations could increase the risk of thyroid dysfunction. In addition to the circadian rhythm, sleep could substantially influence thyroid function, as demonstrated by the analysis of data from a representative population.

## Figures and Tables

**Table 1:** Characteristics of the study participants according to sleep duration.

	Participants Group				Multiple Comparison		
	Short Sleepers *n* = 2097	Normal Sleepers *n* = 2514	Long Sleepers *n* = 334	*p*-Value	1 vs. 2 *p*-Value	2 vs. 3 *p*-Value	1 vs. 3 *p*-Value
Age	46.71 ± 0.35	43.46 ± 0.32	41.17 ± 1.09	<0.0001	<0.0001	0.0489	<0.0001
Sex				0.0808	0.8549	0.0860	0.1782
Male	1085 (53.06)	1307 (54.22)	151 (46.96)				
Female	1012 (46.94)	1207 (45.78)	183 (53.04)				
BMI				<0.0001	0.0001	0.0244	<0.0001
<18.5	69 (2.77)	118 (4.52)	31 (9.32)				
18.5–23	761 (35.27)	1030 (40.55)	133 (41.11)				
23–25	523 (25.89)	582 (23.76)	72 (20.25)				
≥25	744 (36.08)	784 (31.18)	98 (29.32)				
Mean	24.22 ± 0.09	23.67 ± 0.07	23.45 ± 0.28	<0.0001	<0.0001	0.4347	0.0079
Diabetes				0.0442	0.6722	0.0493	0.1913
No	1974 (93.87)	2375 (94.64)	307 (90.71)				
Yes	123 (6.13)	139 (5.36)	27 (9.29)				
Current smoking				0.8232	1.0000	0.9082	0.9165
No	1140 (54.61)	1397 (54.55)	190 (56.52)				
Yes	957 (45.39)	1117 (45.45)	144 (43.48)				
Alcohol consumption				0.0042	0.0077	0.0980	0.8691
No	178 (8.83)	172 (6.56)	32 (10.91)				
Yes (<4 times per month)	856 (40.07)	1128 (45.11)	147 (41.10)				
Yes (≥4 times per month)	1063 (51.10)	1214 (48.33)	155 (47.99)				
Regular exercise *				0.9305	0.9960	0.9762	0.9921
No	1178 (57.36)	1421 (57.02)	187 (58.21)				
Yes	919 (42.64)	1093 (42.98)	147 (41.79)				
Household income level (thousand Korean Won)				0.0002	0.0427	0.0003	0.0406
≤1000/month	309 (13.77)	271 (10.24)	63 (18.98)				
1001–2000/month	524 (24.45)	667 (26.94)	97 (27.88)				
2001–3000/month	611 (29.61)	764 (30.49)	100 (29.96)				
≥3001/month	653 (32.17)	812 (32.34)	74 (23.19)				
Education (years)				<0.0001	0.0063	<0.0001	0.0011
≤6	353 (15.70)	318 (12.86)	61 (19.97)				
7–9	224 (11.37)	216 (8.72)	37 (10.94)				
10–12	790 (37.78)	986 (39.91)	150 (46.84)				
>12	730 (35.15)	994 (38.51)	86 (22.24)				
Free T4	1.23 ± 0.00	1.24 ± 0.00	1.25 ± 0.01	0.2233	0.0985	0.8174	0.3251
Anti-TPO antibody level (IU/mL) **				0.8701	0.9817	0.9838	0.9536
<34	1969 (93.79)	2351 (94.05)	317 (94.58)				
≥34	128 (6.21)	163 (5.95)	17 (5.42)				
Urine iodine				0.0036	0.4242	0.0290	0.0017
Quartile 1	506 (22.96)	661 (25.85)	110 (34.03)				
Quartile 2	529 (25.60)	623 (24.80)	82 (25.32)				
Quartile 3	522 (25.39)	628 (25.71)	69 (18.36)				
Quartile 4	540 (26.05)	602 (23.64)	73 (22.29)				
Working pattern				0.0010	0.1434	0.0055	0.0045
Daytime	1802 (85.88)	2160 (85.56)	263 (78.98)				
Evening	144 (7.51)	208 (8.68)	47 (14.49)				
Nighttime	53 (2.26)	30 (1.36)	11 (3.68)				
Irregular	89 (0.22)	99 (4.41)	10 (2.85)				

Data are presented as *n* (weighted %) and mean ± standard deviation. Statistics were carried out using Rao–Scott chi-square test and ANOVA or *t*-test. Multiple comparison: “1” for “short sleeper; less than 7 h”, “2” for “normal sleepers; 7–8 h”, and “3” for “long sleeper; more than 8 h”. *Regular exercise was defined as strenuous physical activity for more than 20 min per day at least three days per week, moderate physical activity for more than 30 min at least five days per week, or walking for more than 30 min at least five days per week. ** TPO, thyroid peroxidase. BMI, body mass index; T4, tetraiodothyronine.

**Table 2:** The prevalence of subclinical thyroid dysfunctions and mean serum thyrotropin (TSH) levels according to sleep duration.

	Participants Group				Multiple Comparison		
	Short Sleepers *n* = 2097	Normal sleepers *n* = 2514	Long Sleepers *n* = 334	*p*-Value	1 vs. 2 *p*-Value	2 vs. 3 *p*-Value	1 vs. 3 *p*-Value
TSH level (mIU/L)	2.58 ± 0.04	2.59 ± 0.04	2.85 ± 0.14	0.1677	0.7670	0.0688	0.0607
Euthyroidism	1960 (93.10)	2395 (95.06)	306 (91.49)	0.0116	0.0489	0.0411	0.7193
Non-euthyroidism	137 (6.90)	119 (4.94)	28 (8.51)	0.0399	0.0983	0.1360	0.8982
Subclinical hyperthyroidism	63 (3.17)	56 (2.58)	16 (4.47)				
Subclinical hypothyroidism	74 (3.73)	63 (2.35)	12 (4.04)				

Data are presented as n (weighted %) and mean ± standard deviation. Multiple comparison: “1” for “short sleeper; less than 7 h”, “2” for “normal sleepers; 7–8 h”, and “3” for “long sleeper; more than 8 h”. Statistics were carried out using Rao–Scott chi-square test and *t*-test.

**Table 3:** Risk of subclinical thyroid dysfunctions according to sleep duration.

	Subclinical Hyperthyroidism		Subclinical Hypothyroidism	
	Odds Ratio (95% confidence interval)	*p*-Value	Odds Ratio (95% confidence interval)	*p*-Value
Sleep duration				
Crude				
Normal sleepers	reference		reference	
Short sleepers	1.43 (1.07–1.91)	0.0174	1.25 (0.84–1.88)	0.2748
Long sleepers	1.79 (1.12–2.87)	0.0155	1.80 (0.97–3.34)	0.0626
Model 1				
Normal sleepers	reference		reference	
Short sleepers	1.38 (1.02–1.85)	0.0340	1.21 (0.80–1.81)	0.3670
Long sleepers	1.77 (1.11–2.84)	0.0175	1.80 (0.97–3.33)	0.0609
Model 2				
Normal sleepers	reference		reference	
Short sleepers	1.37 (1.02–1.84)	0.0364	1.22 (0.81–1.83)	0.3365
Long sleepers	1.79 (1.12–2.86)	0.0154	1.91 (1.03–3.53)	0.0399
Model 3				
Normal sleepers	reference		reference	
Short sleepers	1.35 (1.00–1.82)	0.0484	1.17 (0.78–1.78)	0.4458
Long sleepers	1.77 (1.11–2.84)	0.0169	1.89 (1.02–3.49)	0.0441

Data are presented as odds ratio (OR) (95% confidence interval (CI)). Statistics were carried out using Logistic regression. Model 1: adjusted by age and sex. Model 2: adjusted by age, sex, BMI, diabetes, alcohol consumption, household income level, education, and urine iodine creatinine ratio. Model 3: adjusted by age, sex, BMI, diabetes, alcohol consumption, household income level, education, urine iodine creatinine ratio, and working pattern.

**Table 4:** Risk of subclinical thyroid dysfunctions according to sleep duration in subjects with anti-TPO antibody less than 34 IU/mL.

	Subclinical Hyperthyroidism		Subclinical Hypothyroidism	
	Odds Ratio (95% confidence interval)	*p*-Value	Odds Ratio (95% confidence interval)	*p*-Value
Sleep duration				
Crude				
Normal sleepers	reference		reference	
Short sleepers	1.47 (1.07–2.02)	0.0188	1.19 (0.78–1.82)	0.4242
Long sleepers	1.78 (1.08–2.94)	0.0232	1.87 (1.00–3.48)	0.0488
Model 1				
Normal sleepers	reference		reference	
Short sleepers	1.41 (1.02–1.95)	0.0362	1.14 (0.75–1.75)	0.5429
Long sleepers	1.77 (1.08–2.91)	0.0240	1.87 (1.01–3.47)	0.0480
Model 2				
Normal sleepers	reference		reference	
Short sleepers	1.41 (1.02–1.96)	0.0375	1.15 (0.75–1.76)	0.5095
Long sleepers	1.83 (1.11–3.02)	0.0187	2.00 (1.08–3.70)	0.0280
Model 3				
Normal sleepers	reference		reference	
Short sleepers	1.39 (1.00–1.92)	0.0527	1.10 (0.72–1.70)	0.6533
Long sleepers	1.81 (1.10–2.99)	0.0205	1.97 (1.06–3.65)	0.0325

Data are presented as OR (95% CI). Statistics were carried out using logistic regression. Model 1: adjusted by age and sex. Model 2: adjusted by age, sex, BMI, diabetes, alcohol consumption, household income level, education, and urine iodine creatinine ratio. Model 3: adjusted by age, sex, BMI, diabetes, alcohol consumption, household income level, education, urine iodine creatinine ratio, and working pattern.

## Supplementary Materials

The following are available online at https://www.mdpi.com/2077-0383/8/11/2010/s1, Table S1: Risk of subclinical thyroid dysfunctions according to sleep duration in evening and nighttime workers, Table S2: Risk of subclinical thyroid dysfunctions according to sleep duration (five subgroups).
