# Supplementary material for: Association between Sleep Duration and Subclinical Thyroid Dysfunction Based on Nationally Representative Data
Source: J Clin Med. 2019 Nov 18;8(11):2010. doi: 10.3390/jcm8112010 (PMC6912782; doi:10.3390/jcm8112010)
Supplement: Supplementary file 1 [file jcm-08-02010-s001.pdf]

Supplementary Tables

Table S1. Risk of subclinical thyroid dysfunctions according to sleep duration in evening and nighttime workers

	Subclinical hyperthyroidism		Subclinical hypothyroidism	
	Odds ratio (95% confidence interval)	*p*-value	Odds ratio (95% confidence interval)	*p*-value
Sleep duration				
Crude				
Normal sleepers	reference		reference	
Short sleepers	1.974 (0.821-4.748)	0.1284	2.994 (0.657-13.649)	0.1562
Long sleepers	2.315 (0.713-7.519)	0.1621	1.961 (0.275-13.986)	0.5010
Model 1				
Normal sleepers	reference		reference	
Short sleepers	2.048 (0.855-4.904)	0.1075	2.969 (0.650-13.564)	0.1600
Long sleepers	2.005 (0.629-6.389)	0.2388	1.984 (0.296-13.286)	0.4796
Model 2				
Normal sleepers	reference		reference	
Short sleepers	1.995 (0.817-4.875)	0.1293	3.655 (0.672-19.871)	0.1333
Long sleepers	1.869 (0.617-5.666)	0.2682	1.817 (0.328-10.075)	0.4937
Model 3				
Normal sleepers	reference		reference	
Short sleepers	2.009 (0.799-5.053)	0.1379	3.426 (0.634-18.500)	0.1520
Long sleepers	1.872 (0.615-5.699)	0.2692	1.885 (0.356-9.987)	0.4555

Data are presented OR (95% CI).

Statistics were carried out using Logistic regression.

Model 1: adjusted by age and sex

Model 2: adjusted by age, sex, BMI, diabetes, alcohol consumption, household income level, education, and urine iodine creatinine ratio

Model 3: adjusted by age, sex, BMI, diabetes, alcohol consumption, household income level, education, urine iodine creatinine ratio and working pattern

Table S2. Risk of subclinical thyroid dysfunctions according to sleep duration (5 subgroups)

	Subclinical hyperthyroidism		Subclinical hypothyroidism	
	Odds ratio (95% confidence interval)	*p*-value	Odds ratio (95% confidence interval)	*p*-value
Sleep duration				
Crude				
Extreme short sleepers	2.375 (1.230-4.585)	0.0101	2.424 (1.016-5.784)	0.0460
Mild short sleepers	1.352 (1.005-1.818)	0.0466	1.159 (0.761-1.766)	0.4919
Normal sleepers	reference		reference	
Mild long sleepers	1.779 (1.089-2.907)	0.0216	1.783 (0.930-3.418)	0.0815
Extreme long sleepers	2.021 (0.535-7.630)	0.2985	2.122 (0.453-9.954)	0.3392
Model 1				
Extreme short sleepers	2.065 (1.051-4.057)	0.0354	2.146 (0.863-5.334)	0.1002
Mild short sleepers	1.319 (0.979-1.778)	0.0688	1.128 (0.740-1.721)	0.5741
Normal sleepers	reference		reference	
Mild long sleepers	1.758 (1.077-2.872)	0.0242	1.785 (0.935-3.409)	0.0789
Extreme long sleepers	2.010 (0.523-7.732)	0.3092	2.101 (0.445-9.905)	0.3476
Model 2				
Extreme short sleepers	2.100 (1.057-4.170)	0.0341	2.316 (0.943-5.689)	0.0670
Mild short sleepers	1.312 (0.972-1.770)	0.0758	1.134 (0.743-1.732)	0.5585
Normal sleepers	reference		reference	
Mild long sleepers	1.781 (1.094-2.902)	0.0205	1.911 (1.001-3.647)	0.0495
Extreme long sleepers	2.124 (0.526-8.580)	0.2898	2.166 (0.435-10.784)	0.3446
Model 3				
Extreme short sleepers	2.121 (1.063-4.229)	0.0329	2.315 (0.933-5.744)	0.0702
Mild short sleepers	1.289 (0.953-1.743)	0.0999	1.087 (0.707-1.672)	0.7040
Normal sleepers	reference		reference	
Mild long sleepers	1.763 (1.082-2.873)	0.0229	1.887 (0.986-3.611)	0.0550
Extreme long sleepers	2.159 (0.531-8.784)	0.2819	2.212 (0.444-11.013)	0.3318

Data are presented OR (95% CI).

Statistics were carried out using Logistic regression.

Model 1: adjusted by age and sex

Model 2: adjusted by age, sex, BMI, diabetes, alcohol consumption, household income level, education, and urine iodine creatinine ratio

Model 3: adjusted by age, sex, BMI, diabetes, alcohol consumption, household income level, education, urine iodine creatinine ratio and working pattern
